# Inhibitors of SARS-CoV-2 PLpro

**DOI:** 10.3389/fchem.2022.876212

**Published:** 2022-04-26

**Authors:** Dale J. Calleja, Guillaume Lessene, David Komander

**Affiliations:** ^1^ Walter and Eliza Hall Institute, Parkville, VIC, Australia; ^2^ Department of Medical Biology, University of Melbourne, Melbourne, VIC, Australia; ^3^ Department of Pharmacology and Therapeutics, The University of Melbourne, Melbourne, VIC, Australia

**Keywords:** antiviral drug discovery, SARS-CoV-2, COVID-19, papain like protease (PLpro), Nsp3, GRL-0617, structure-activity relationship (SAR), medicinal chemistry

## Abstract

The emergence of SARS-CoV-2 causing the COVID-19 pandemic, has highlighted how a combination of urgency, collaboration and building on existing research can enable rapid vaccine development to fight disease outbreaks. However, even countries with high vaccination rates still see surges in case numbers and high numbers of hospitalized patients. The development of antiviral treatments hence remains a top priority in preventing hospitalization and death of COVID-19 patients, and eventually bringing an end to the SARS-CoV-2 pandemic. The SARS-CoV-2 proteome contains several essential enzymatic activities embedded within its non-structural proteins (nsps). We here focus on nsp3, that harbours an essential papain-like protease (PLpro) domain responsible for cleaving the viral polyprotein as part of viral processing. Moreover, nsp3/PLpro also cleaves ubiquitin and ISG15 modifications within the host cell, derailing innate immune responses. Small molecule inhibition of the PLpro protease domain significantly reduces viral loads in SARS-CoV-2 infection models, suggesting that PLpro is an excellent drug target for next generation antivirals. In this review we discuss the conserved structure and function of PLpro and the ongoing efforts to design small molecule PLpro inhibitors that exploit this knowledge. We first discuss the many drug repurposing attempts, concluding that it is unlikely that PLpro-targeting drugs already exist. We next discuss the wealth of structural information on SARS-CoV-2 PLpro inhibition, for which there are now ∼30 distinct crystal structures with small molecule inhibitors bound in a surprising number of distinct crystallographic settings. We focus on optimisation of an existing compound class, based on SARS-CoV PLpro inhibitor GRL-0617, and recapitulate how new GRL-0617 derivatives exploit different features of PLpro, to overcome some compound liabilities.

## Introduction

The COVID-19 pandemic and its causative coronavirus (CoV), SARS-CoV-2 continues to wreak havoc in many countries. The rate at which global disruption took place and the continual challenges presented to countries globally, and to people individually, lack comparisons to any other events in past generations. Science stepped up to the challenge, and provided a remarkable response, and solutions, saving lives within a very short timeframe, through implementation of public health measures and even more strikingly, through rapid development of vaccines. Considering that global or local measures on how to deal with a pandemic were by-and-large non-existent prior to 2020, this has been an extraordinary feat. At the same time, the latest emergence of the SARS-CoV-2 Omicron variants (B.1.1.529) serves as a reminder that the pandemic is far from over and COVID-19 continues to kill people daily. It is now widely accepted that it is essential to not only provide protection but also improve treatment options for individuals in which SARS-CoV-2 infection may lead to severe illness, hospitalisation, and death.

One form of such treatments emerges from exploiting the accumulated knowledge around the viral pathogens, in particular Coronaviruses (Almeida et al., 1968; Hartenian et al., 2020; V’kovski et al., 2020). The first Coronaviruses, B814 (Tyrrell and Bynoe, 1965), 229E (Hamre and Procknow, 1966) and OC43 (McIntosh et al., 1967) were identified in the late 1960s and CoV infections in humans are quite common, mostly leading to mild symptoms, and were therefore largely neglected in the wider population and in the scientific community (Paules et al., 2020). The first widely noted, deadly-to-human, CoV was SARS-CoV (Drosten et al., 2003; Ksiazek et al., 2003; Peiris et al., 2003), that caused an epidemic in 2003. Since then, new CoVs were identified frequently, to include NL63-CoV (Hoek et al., 2004), HKU1-CoV (Woo et al., 2005), MERS-CoV (Drosten et al., 2003; Boheemen et al., 2012), and then in December 2019, SARS-CoV-2 (Wu F. et al., 2020; Lu et al., 2020; Zhou et al., 2020; Zhu et al., 2020). Deadly (but not exceedingly infectious) viruses such as SARS-CoV and MERS-CoV clearly showed their pathological potential (Paules et al., 2020). Predicting the origin for SARS-CoV-2 or that of the next CoV remains difficult (Andersen et al., 2020; Zhang and Holmes, 2020; Holmes et al., 2021; Koopmans et al., 2021) largely because their use of discontinuous transcription for viral replication, which allows for a high rate of recombination between different species (Sola et al., 2015), a feature also noted in SARS-CoV-2 (Kim et al., 2020).

Despite this considerable sequence diversity within CoVs, the overall makeup of the CoV genome is identical and offers numerous functional access points for interference. The main steps in a viral life cycle include entry into the host cell, followed by release of the viral genome that is then translated by the host cells’ ribosomes (Hartenian et al., 2020; V’kovski et al., 2020). The translation products are polyproteins that require processing, self-cleavage, into individual functional proteins that either have structural roles in forming viral particles, or non-structural roles such as facilitating replication of the viral genome. Assembly of new viral particles and exocytosis of the mature virions through the formation of double membraned vesicles (DMVs) completes the viral life cycle (Hartenian et al., 2020; V’kovski et al., 2020). The roles of each of the viral structural/accessory protein and non-structural proteins (nsps) have been studied extensively (Hartenian et al., 2020; V’kovski et al., 2020; Chazal, 2021). Targeting essential steps early in the process of viral replication has been the most successful strategy to stop CoV infection.

Creation of 16 individual nsps and (re)assembly of a subset to generate of a functional viral replicase responsible for carbon-copying viral genetic material (Subissi et al., 2014; Malone et al., 2022) are the key upstream steps conserved in all CoVs, and have been the focus of antiviral drug discovery (Malone et al., 2022). The drug Remdesivir, first developed against the replicase of the Hepatitis C virus and later found to also target the Ebola virus (*Zaire Ebolavirus* of the *Filoviridae* family unrelated to CoVs) (Warren et al., 2016; Tchesnokov et al., 2019) and zoonotic CoVs (Sheahan et al., 2017; Agostini et al., 2018; Brown et al., 2019; Eastman et al., 2020), had been the first and for some time only FDA approved small molecule treatment of COVID-19 (Beigel et al., 2020; FDA, 2020; Goldman et al., 2020; Spinner et al., 2020). Its utility has since been refuted by the World Health Organisation (WHO) (Consortium et al., 2020), and Remdesivir is no longer recommended for use in the clinic (WHO, Therapeutics and COVID-19 Living Guideline, 03 March 2022 Update). Ridgeback Therapeutics and Merck developed and clinically tested Molnupiravir, an oral SARS-CoV-2 replicase inhibitor, which was approved by the FDA in December 2021, and which was initially reported to show 50% efficacy against hospitalisation or death in COVID-19 patients (Bernal et al., 2021; FischerII et al., 2021). Molnupiravir benefitted from earlier work on the Venezuelan equine encephalitis virus (an *Alphavirus* of the *Togoviridae* family unrelated to CoVs) before its focus was shifted towards testing against CoVs. Molnupiravir inhibits viral replication in mouse models of SARS-CoV and MERS-CoV (Sheahan et al., 2020) and of SARS-CoV-2 in ferrets (Cox et al., 2021). Unlike Remdesivir which acts to terminate chain elongation during viral replication (Tchesnokov et al., 2019; Gordon C. J. et al., 2020), Molnupiravir induces countless mutations in the nascent RNA strand, effectively causing the virus to mutate itself to death (Gordon et al., 2021; Willyard, 2021). The efficacy of Molnupiravir has since been revised to 30% (Kozlov, 2021) and concerns were raised that Molnupiravir may induce mutations in patient DNA (Zhou et al., 2021). Despite these issues, Molnupiravir was FDA-approved in December 2021 for emergency use in at-risk adults (FDA, 2021).

The steps prior to assembly of the viral replicase present a further, exploitable viral Achilles Heel. Cleavage of the viral polyprotein is facilitated by two viral proteases that perform specific cleavage events to release individual nsps (Hartenian et al., 2020; V’kovski et al., 2020). The first protease is a papain-like protease domain, PLpro, within the large nsp3 protein, which is responsible for cleaving sequences between nsp1 and nsp2, nsp2 and nsp3, and nsp3 and nsp4 (Harcourt et al., 2004). The second protease is the main protease or Mpro encoded by nsp5, which is responsible for cleaving the polyproteins at 11 further sites to release the remaining 12 nsps (Fan et al., 2004). Both activities are essential for viral replication and therefore, both PLpro and Mpro are prime drug targets in CoVs, including SARS-CoV-2 (Hilgenfeld, 2014; Báez-Santos et al., 2015; Lei et al., 2018). Indeed, in late 2021, an oral Mpro inhibitor termed Nirmatrelvir successfully completed clinical trials (clinical trial identifier NCT04960202, NCT05011513) (Owen et al., 2021), and became the third FDA approved small molecule drug against SARS-CoV-2, reaching 89% efficacy in clinical settings against severely ill patients (NCT04960202). The development of Nirmatrelvir benefitted from corporate memory available within Pfizer, who restarted earlier drug discovery efforts targeting SARS-CoV Mpro, leading to a record-breaking timeline for the development of a first-in-human approved small molecule drug (Owen et al., 2021). Mpro inhibitors are further reviewed in (Cui et al., 2020) and (Mengist et al., 2021).

In this Review, we focus on inhibiting PLpro, the remaining highly attractive and druggable target in CoVs (also recently reviewed in (Jiang et al., 2022)). Like Mpro, PLpro is a Cysteine protease, however both enzymes are structurally unrelated and cleave distinct sequences. Moreover, while Mpro appears to have only a small number of non-viral host substrates (Gordon DE. et al., 2020), PLpro moonlights as a potent regulator of host cell signalling processes for its ability to cleave ubiquitin and ubiquitin-like interferon-stimulated gene (ISG)15 posttranslational modifications. The latter deubiquitinase/DUB and deISGylase activities enable use of methodologies, tools and assays developed for current drug discovery efforts that target human DUBs, which have emerged as drug targets for a variety of conditions from cancer (Fraile et al., 2012) to neurodegenerative diseases (Schmidt et al., 2021); however to date only few DUB inhibitors have entered into clinical trials and none have been approved for use in humans (Schauer et al., 2020).

Despite cleaving ubiquitin and ISG15, PLpro is structurally dissimilar to human DUBs. Nevertheless, some parallels to human ubiquitin specific proteases (USPs) can be drawn (Mevissen and Komander, 2016). These similarities became apparent through the initial structural studies on SARS-CoV and MERS-CoV PLpro, reported over the last 15 years in a host of comprehensive studies by the Mesecar, Pegan, Lima and other labs (Harcourt et al., 2004; Barretto et al., 2005; Lindner et al., 2005; Ratia et al., 2006; Lee et al., 2015; Békés et al., 2016) The works explained biochemistry and substrate binding for DUB and ISG15 cleavage in molecular detail, and highlighted variations on the theme of PLpro activities present between CoVs. A further highly significant insight into SARS-CoV in particular, was the drugability of PLpro by small molecule inhibitors. Influential studies in 2008 and 2010 (Ratia et al., 2008; Ghosh et al., 2009, 2010) reported on two distinct chemical series, disclosed some of the first co-crystal structures of a DUB inhibited by small molecules, and presented considerable SAR data on each series of sub-µM inhibitors. A follow up study in 2014 then improved on the metabolic stability of these compounds, as well as presenting a co-crystal structure of SARS-CoV PLpro in complex with compound ***3k*** (Báez-Santos et al., 2014) (also see our associated manuscript, Calleja et al., in this issue).

In 2020, many research groups including ours quickly appreciated the high similarity at the sequence and structural level between SARS-CoV and SARS-CoV-2 PLpro, and excitingly, it was found that SARS-CoV PLpro inhibitors were able to also inhibit SARS-CoV-2 PLpro with almost identical activity profiles (Freitas et al., 2020; Klemm et al., 2020; Shin et al., 2020). We focussed our efforts on a series of compounds based on a central piperidine chemical scaffold, the most developed and potent SARS-CoV PLpro inhibitors available, and we showed that ***5c***, a compound targeting SARS-CoV PLpro (Báez-Santos et al., 2014), was a potent *in vitro* inhibitor with antiviral activity in a cell-based SARS-CoV-2 infection model (Klemm et al., 2020). Our latest data on this series of compounds is discussed in Calleja et al. elsewhere in this issue. Many other groups focussed on a second chemical scaffold, exemplified by a compound named ***GRL-0617*** (Ratia et al., 2008; Ghosh et al., 2009). A wealth of data reported within the last 2 years, has since provided structural data to the Protein Data Bank (PDB) on numerous co-crystal structures for this scaffold. We here review the ideas and progress revealed in recent publications, starting by a discussion of the manifold efforts to inhibit PLpro *via* available medicines, in drug repurposing campaigns.

## Drug Repurposing

Successful drug discovery efforts beginning from hit discovery can take decades and billions of dollars of investment. For PLpro and Mpro, earlier campaigns may (and in case of Mpro, did) accelerate such timelines considerably. However, when the pandemic hit, drug repurposing (or repositioning) was heralded as a way to fast track translation, with the assumption that within the available, but somewhat limited, collection of drugs approved for use in humans, some may have off-label uses for COVID-19. This initially offered high hopes of success, perhaps for the wrong reasons (Begley et al., 2021). In fact, there are only very few examples of successful drug repurposing (Begley et al., 2021).

Nonetheless, many drug repurposing libraries exist that include FDA-approved small molecules, but also compounds that have undergone late-stage preclinical development or entered clinical trials. Such commonly used libraries include the ReFRAME (Repurposing, Focused Rescue and Accelerate MedChem) (Janes et al., 2018), the Sigma Aldrich LOPAC^®^1,280 (List Of Pharmacologically Active Compounds) and other designer libraries, hand-selected by researchers. Many High Throughput Screening (HTS) campaigns for drug repurposing involved either target-based assays or phenotypic screening to identify drugs for treating COVID-19.

## Biochemical Assay Design for PLpro HTS—Technical Considerations

High throughput screens for viral proteases are typically activity based *in vitro* screens exploiting knowledge of protease target sequence(s). Most screens are based on fluorescence spectroscopy, where a fluorophore is conjugated to a peptide substrate based on its natural cleavage sequence. For PLpro, an additional route exploited its DUB activity. Activity-based DUB assays measure cleavage of a folded protein, ubiquitin, at its C-terminus, and enzymes such as PLpro not only comprise binding elements for the C-terminal sequence (LRLRGG) of ubiquitin or ISG15, but also contain a binding surface that covers a significant portion of the 8,000 Å^2^ ubiquitin surface. Indeed, it has been shown that cleavage of a fluorophore conjugated to ubiquitin is >10,000-fold more efficient than cleavage of a peptide-only substrate. The presence of ubiquitin likely orients and stabilises the target peptide in the catalytic cleft, contributing to catalytic efficiency of the cleavage reaction (Dang et al., 1998).

For both types of assays, hydrolysis of the substrate peptide or ubiquitin releases the fluorophore and generates a fluorescent signal indicative of enzyme activity that can be measured. 7-Amido-4-Methylcoumarin (AMC) (Dang et al., 1998) or a disubstituted Rhodamine moiety (Rh110) (Hassiepen et al., 2007) are well established fluorophores for measuring enzymatic cleavage of ubiquitin substrates. AMC however, holds a significant disadvantage as its excitation wavelength is in the UV range (341 nm). While most HTS screens will use substrate concentrations of around or below the K_m_ to identify competitive inhibitors, in the situation of highly active enzymes (such as in the case of most viral proteases), the K_m_ for the enzyme is low and the concentration of compound required to detect inhibition is high enough such that compounds may absorb UV light, and thus run the risk of being potentially identified as false positives. The Rh110 moiety is advantageous in assaying for potential PLpro inhibitors, as it alleviates many of the AMC limiting factors, as well as provides a broader dynamic range. Pan Assay Interfering Compounds (PAINS) (Baell and Holloway, 2010) are a key challenge in any HTS campaign and may have been overlooked in some studies reported during the pandemic. Their identification can be difficult and can distract from bona fide hits. A key consideration in the identification of viral protease inhibitors is the design of suitable and ideally meaningful secondary assays and counter screens, to test for direct binding of compounds to target, and to assess specificity by testing compounds on other protease(s) (structurally similar or dissimilar). Indeed, from our experience, many small molecule “hits” from an enzymatic assay fail to confirm in orthogonal assays such as SPR and ITC, and therefore stringent criteria for activity and binding in orthogonal assays are essential for DUB drug discovery programs. Notably, the results reported over the last 2 years discussed below, have often failed to include careful evaluation and validation of the hits arising from HTS campaigns, and most repurposing campaigns did not provide orthogonal analysis of their “hit” compounds (summarised in Table 1). Moreover, while *in vitro* assays are often performed against the PLpro domain in isolation, a further, important orthogonal assay expands on this work to test the activity of PLpro inhibitors towards full-length nsp3 as expressed by the virus. A nice advance for testing compounds against full-length nsp3, are cell-based activity assays based on the FlipGFP reporter (Zhang et al., 2019), an assay shown to be successful in detecting the inhibition of both Mpro (Froggatt et al., 2020) and PLpro (Ma et al., 2021).

**TABLE 1:** Summary of reported drug repurposing attempts against SARS-CoV-2 PLro.

Name	Chemical Structure(s)	Current Clinical Use(s)	Primary assay	Orthogonal Assay(s)	Counter Screen(s)	Comments	References(s)
Tanshinone and derivatives							
Sodium Tanshinone IIA Sulfonate	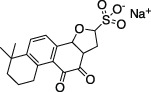	Hypertension, Myocardial infarction, coronary artery disease	Z-ALKGG-AMC	SPR (K_D_ of 61 µM)	No	- Dosage used substantially higher than the clinic	Xu et al. (2021)
			ISG15-FITC (FP assay)			- K_D_ measured of 61 µM	
Cryptotanshinone	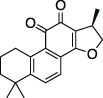	Immunosuppressant, anticancer treatment, vasodilator	Plaque reduction assay	No	No	- Not tested against full Ub substrate	Lim et al. (2021); Zhao et al. (2021)
						- No assay to measure direct binding	
						- More potent in cells than in biochemical assay, suggests cytotoxicity	
Tanshinone I	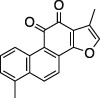	Oncolytic treatment, chemopreventative agent	Abz-FTLKGGAPTKVT-DNP (FRET)	No	No	- Not tested against full Ub substrate	
Tanshinone IIA	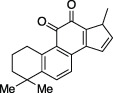	Oncolytic drug, vasodilator, treatment of stroke				- No conclusive follow up assays	Lim et al. (2021)
						- Known inducer of apoptosis Fang et al. (2021)	
HCV Drugs							
Asunaprevir	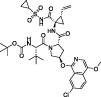	Hepatitis C Virus (HCV), NS3/4 serine protease inhibitor	Z-RLRGG-AMC	No	No	- Extremely low starting IC_50_ (54 µM)	Anson et al. (2020)
						- Optimised for a very different protease	
Simeprevir	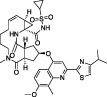		Phenotypic screen	No	No	- Entirely phenotypic screen	Anson et al., (2020); Gammeltoft et al. (2021)
						- Often a sharp decline in cell viability, indicating just lagging in dying of virus	
Vaniprevir/Simeprevir	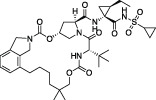		Z-RLRGG-AMC	No	No	- Follow up assays solely synergistic phenotypic screens	
Others							
Famotidine	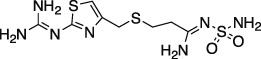	H2AR agonist – stomach and intestinal ulcers	Z-ALKGG-AMC	No	No	- Dosing used far exceeds its intended indication	Wu et al., 2020a; Kandeel et al., 2020)
			ISG15-FITC (FP assay)			- Refuted in a recent *in vitro* study Loffredo et al. (2021)	
Ebselen	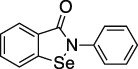	Meniere’s disease and hearing loss	Ub-AMC	No	No	- Se is highly reactive against cysteines	Sargsyan et al. (2020); Weglarz-Tomczak et al. (2021)
						- Only showed inhibitory activity after a prolonged incubation	
Disulfiram	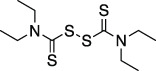	Anti-cancer agent	Abz- FTLKGGAPTKVT-DNP (FRET)	No	No	- Likely nonspecific to all zinc finger containing proteins (including other DUBs)	Sargsyan et al. (2020)
Acriflavine	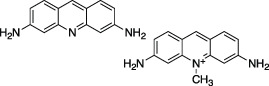	Nil	Z-RLRGG-AMC	No	No	- DNA intercalating agent	Napolitano et al. (2022)
						- Biological targets are unclear	
Repurposed “lead” compounds							
*GRL-0617*	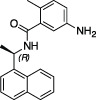	Nil	Varies (see Table 2)	Yes	Yes	- Repurposed early stage compound optimised for SARS-CoV	Shin et al. (2020) and see Table 2
*5c*	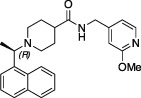	Nil	Ub-Rh110	Yes	Yes		Klemm et al. (2020); Shan et al. (2021)
*3k*	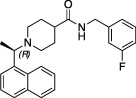	Nil	Ub-Rh110	Yes	Yes		Calleja et al. in this issue

## Critical Assessment of Examples of Drug Repurposing “Hits”

Numerous drug repurposing studies reported putative PLpro inhibitors. We do not discuss a large set of *in silico* studies based on compound docking as they lack binding or inhibition data, but focus on those studies where biochemical data was obtained. For repurposing using a PLpro directed assay, distinct HTS libraries were used, including the ReFRAME library (Smith et al., 2020; Redhead et al., 2021) and Calleja et al. in this issue of Frontiers In Chemistry, LOPAC1280 (Klemm et al., 2020), ApexBio FDA approved drug library (Xu et al., 2021), the Pathogens Box Library from Medicines for Malaria Venture (Smith et al., 2020), and libraries of FDA approved drugs and natural products from Selleck Chem (Zhao et al., 2021). In addition, some reports used custom, hand-selected compound libraries (Anson et al., 2020; Lim et al., 2021). Each effort yielded putative PLpro inhibitors, summarised in Table 1 and discussed below.

### Tanshinone and Derivatives

Multiple studies have reported Tanshinone derivatives as inhibitors of SARS-CoV-2 viral replication with PLpro as the proposed target (Lim et al., 2021; Xu et al., 2021; Zhao et al., 2021). Tanshinones are compounds found naturally in the plant *Salvia miltiorrhiza* commonly used in Chinese medicine. It is thought that many naturally bioactive molecules are inhibitors of the CoV proteases (Benarba and Pandiella, 2020; Khare et al., 2020; Chen W. et al., 2021) and the fact that Tanshinones have appeared in multiple independent studies, including that for SARS-CoV, could indicate it is a true inhibitor of PLpro. A follow-up study (Ma and Wang, 2022) thoroughly tested the reported Tanshinone based compounds in a cellular assay, and invalidated earlier findings by showing that compound activity was much lower than reported. Moreover, many of the aforementioned studies lack effective orthogonal assays demonstrating a direct interaction between these compounds and PLpro. Tanshinone derivatives feature many chemical liabilities; two reactive ketone groups and an orthoquinone moiety are known to be redox substrates. Redox cycling compounds have been shown to generate hydrogen peroxide (H_2_O_2_) in the presence of reducing agents found in most protein buffers (Johnston, 2011). The presence of a strong oxidant such as H_2_O_2_ in biochemical screening assays could interfere with assay readouts and/or would likely irreversibly oxidise the catalytic cysteine of PLpro and render the protein inactive–with the causative compounds appearing as false positives. On the other hand, it is interesting that only Tanshinone derivatives with a naphthalene group (Tanshinone I) were able to inhibit DUB activity (Park et al., 2012), as this chemical group features strongly in known PLpro inhibitors (see below). Lack of evidence for direct binding, but more concerningly, no assessment of off-target effects and/or cellular toxicity, make this inhibitor class an unlikely contender for a useful PLpro-based drug.

### Hepatitis C Drugs Asunaprevir, Simeprivir and Grazoprevir

Another set of known drugs gaining traction for use as COVID treatment came from a boutique library of FDA approved Hepatitis C virus (HCV) drugs. Asunaprevir, Simeprivir and Grazoprevir (Anson et al., 2020) are nanomolar HCV NS3/NS4 serine protease inhibitors and were suggested to also target SARS-CoV-2 PLpro. One report (Gammeltoft et al., 2021) showed that combination treatment of Remdesivir with either Simeprevir, Grazoprevir or Paritaprevir was able to reduce viral titers in an *in vitro* viral replication assay. However, it was not clear whether these effects were synergistic nor did the work specify a putative target for the drugs. As for many identified treatments of viral replication, there was also a delicate balance between effectiveness and cytotoxicity at the concentrations required. The resulting poor Selectivity Index of the compounds suggests that use in humans would be challenging. Another study (Bafna et al., 2021) showed promising synergistic data for Paritaprevir and Grazoprevir in combination with Remdesivir. The hypothesis that PLpro is the target of the drugs was undermined by data showing only a weak inhibitory activity (20–25%) against PLpro in an AMC assay (Bafna et al., 2021). Again, this suggested that compound efficacy is most likely due to off-target effects. As before, incomplete reports lacking thorough biochemical investigation combined with the need for high, likely toxic, dosages of these compounds raises questions about their use as effective treatments for COVID-19 and for their specificity towards PLpro. Moreover, with a starting IC_50_ of 54 µM (Anson et al., 2020) for Asunaprevir, medicinal chemistry to improve these already complex compounds would prove challenging.

### Famotidine

Famotidine entered the repurposing stage after reports of a retrospective study in China highlighted patients taking the drug exhibited improved clinical outcomes–the data associated with these reports remain unpublished. Famotidine, marketed as Pepcid^®^, is an FDA approved histamine H2 receptor antagonist prescribed to treat heartburn. Two retrospective studies later conducted in the US also confirmed similar findings (Freedberg et al., 2020; Mather et al., 2020), however, like most reports for repurposed drugs, the mechanism of action underlying the observed beneficial effects remain ill-defined (Mura et al., 2021). One computational study suggested Mpro (Wu C. et al., 2020) as the target, while another suggested PLpro (Kandeel et al., 2020). Experimental data found no evidence for Famotidine to bind or inhibit PLpro *in vitro,* and the compound was unable to inhibit SARS-CoV-2 replication in a cellular infection model (Loffredo et al., 2021). It is possible that the observed clinical benefits of Famotidine resulted from its primary function as a histamine H2A receptor antagonist (Malone et al., 2021). Nevertheless, the DrugBank (Wishart et al., 2006, 2017) database indicates that Famotidine has entered a number of clinical trials for the treatment of COVID-19 (DrugBank Accession Number DB00927); the results of the studies are yet to be released.

### Cysteine Modifying Compounds, Ebselen and Disulfiram

Ebselen and Disulfiram are two cysteine reactive compounds, which were previously identified as inhibitors of SARS-CoV and MERS-CoV PLpro (Lin et al., 2018) and more recently of SARS-CoV-2 PLpro and Mpro (Jin et al., 2020; Amporndanai et al., 2021). For PLpro, it was proposed that enzyme inhibition occurs by inducing oxidation of the catalytic cysteine, or one of the cysteines found in the zinc finger domain, thereby reducing PLpro stability (Sargsyan et al., 2020; Weglarz-Tomczak et al., 2021). Interestingly, similar reactivity with the catalytic cysteine of Mpro was recently observed, sparking optimistic hopes of a multitarget drug. However, Ebselen is a Selenium containing promiscuous cysteine protease inhibitor (Ma et al., 2020). A recent structure of Ebselen in complex with PLpro C111S mutant (PDB 7M1Y) showed that Ebselen binds distally from both the active site and the zinc binding “Fingers” domain (unpublished). Indeed, the lack of specificity (Ma et al., 2020), common occurrence as a false-positive hit in HTS campaigns, and promiscuous and likely toxic nature of the compounds make such broad oxidants questionable for clinical development towards a viral protease.

### Acriflavine

Acriflavine (ACF), published as a low µM (IC_50_) PLpro inhibitor, was another drug identified with potential for repurposing. ACF showed promising antiviral activity in a number of different cell lines though it was unable to rival Remdesivir in blocking viral replication in the lungs of K18-ACE2 mice (Napolitano et al., 2022). The published structure of PLpro in complex with a component of ACF, Proflavine (PDB 7NT4), indicates Proflavine is the active component inhibiting PLpro. Proflavine exists as a low level DNA intercalating agent (and hence a possible carcinogen) (Gatasheh et al., 2017), which likely elicits an antiviral response *via* premature activation of the cGAS-STING pathway (Pépin et al., 2017). Confounding its on-target specificity, ACF has been shown to reduce tumour growth by directly inhibiting HIF-1a dimerization (Lee et al., 2009). ACF appears to be under investigation for its use in treating diseases such as cancer (Cheloni et al., 2017; Mangraviti et al., 2017; Nehme et al., 2020) and malaria (Dana et al., 2014); or as a topical ointment for treating chronic urinary tract infections (Gama et al., 2020). The myriad of indications points towards the polypharmacology of Acriflavine and it is important to note that this compound is not currently approved by the FDA for any of these indications.

## Phenotypic Screens

In addition to PLpro targeted *in vitro* screens, numerous phenotypic screens, measuring the impact of available drugs on SARS-CoV-2 replication in cells, have been performed and reported. While simple conceptually, phenotypic screens can be more complex than biochemical assays and require careful target identification and validation studies to fully elucidate the mechanism underpinning the cellular effect. Phenotypic screen also do not alleviate the need for extensive medicinal chemistry (Moffat et al., 2017). As a result, many compounds dubbed as having potential for repurposing (Riva et al., 2020) have not been further explored. Several detailed reviews recently covered the various drug repurposing attempts for SARS-CoV-2 (Guy et al., 2020; Santos et al., 2020; Ng et al., 2021).

Most prominently, phenotypic screens were performed using a number of known libraries such as the ReFRAME library (Riva et al., 2020), or those from the National Centre for Advancing Translational Sciences (NCATS) (Chen C. Z. et al., 2021), and some of these studies suggested some candidate drugs that inhibited SARS-CoV-2. However, the target of these drugs in a phenotypic screen could either be a viral protein, or a host protein essential for the virus. Importantly, from a PLpro targeting perspective, there was no overlap between putative PLpro inhibitors from *in vitro* studies, and compounds derived from phenotypic screens. This suggested that identified PLpro targeting compounds were not active in phenotypic screens and that compounds derived from phenotypic screens were unlikely to act through PLpro.

### Conclusion for Part I: Drug Repurposing Remains a Complex and Challenging Approach

At the start of the pandemic, without vaccination or active antiviral drugs, drug repurposing was heralded as a silver bullet, and repurposing campaigns were deemed quick-and-easy ways to produce clinic-ready anti-virals. This was ill-considered as outlined recently (Begley et al., 2021), since the development process for any drug remains complex with many ethical and financial considerations such as intellectual property, clinical equipoise and understanding of the drug in a new disease context. In the rare cases where a drug has been repurposed, considerable pre-clinical work is still needed prior to clinical studies. Another concept that seems underappreciated is that drugs are often designed to be very specific modulators of their target proteins. It is therefore highly unlikely that the same compound will have a similar level of potency against an unrelated target. As a consequence, it is likely that significant medicinal chemistry efforts would still be required.

Still, in the DrugBank database, there are currently over 3,000 clinical trials directed towards repurposing efforts for the treatment of COVID-19. Following three separate clinical trials, Remdesivir is currently the only repurposed drug to receive FDA approval for treating COVID-19 (Beigel et al., 2020; FDA, 2020; Goldman et al., 2020; Spinner et al., 2020). This is not surprising, as the mechanism behind viral RNA-dependent-RNA polymerases (RdRp) are inherently conserved. Remdesivir is a nucleoside analogue, required by all viruses to replicate, and initially it held promise as a broad-spectrum antiviral medication. However, despite the apparent theoretical similarities, even Remdesivir failed to be an efficacious drug against COVID-19 and would likely require re-development to optimise it for the SARS-CoV-2 Replicase.

“Lead repurposing” however, has nicely worked for SARS-CoV-2 PLpro. We and others showed that early-stage inhibitors developed for SARS-CoV PLpro are also efficacious against SARS-CoV-2 PLpro. The two most potent of these, ***5c*** and ***GRL-0617***, originated from earlier high throughput screening campaigns followed up by substantial structure guided medicinal chemistry efforts (Ratia et al., 2008; Ghosh et al., 2009; Báez-Santos et al., 2014). While development of SARS-CoV PLpro inhibitors stopped well short of clinical studies, both series have been further advanced against SARS-CoV-2. The efforts toward repurposing outlined above are summarised in Table 1 and we further discuss our own data on ***5c*** series of compounds in the associated research paper (Calleja et al., this issue). Here, we focus on latest reported developments for ***GRL-0617*** series compounds.

### Structural Biology Enables SARS-CoV-2 PLpro Drug Discovery

**FIGURE 1:**
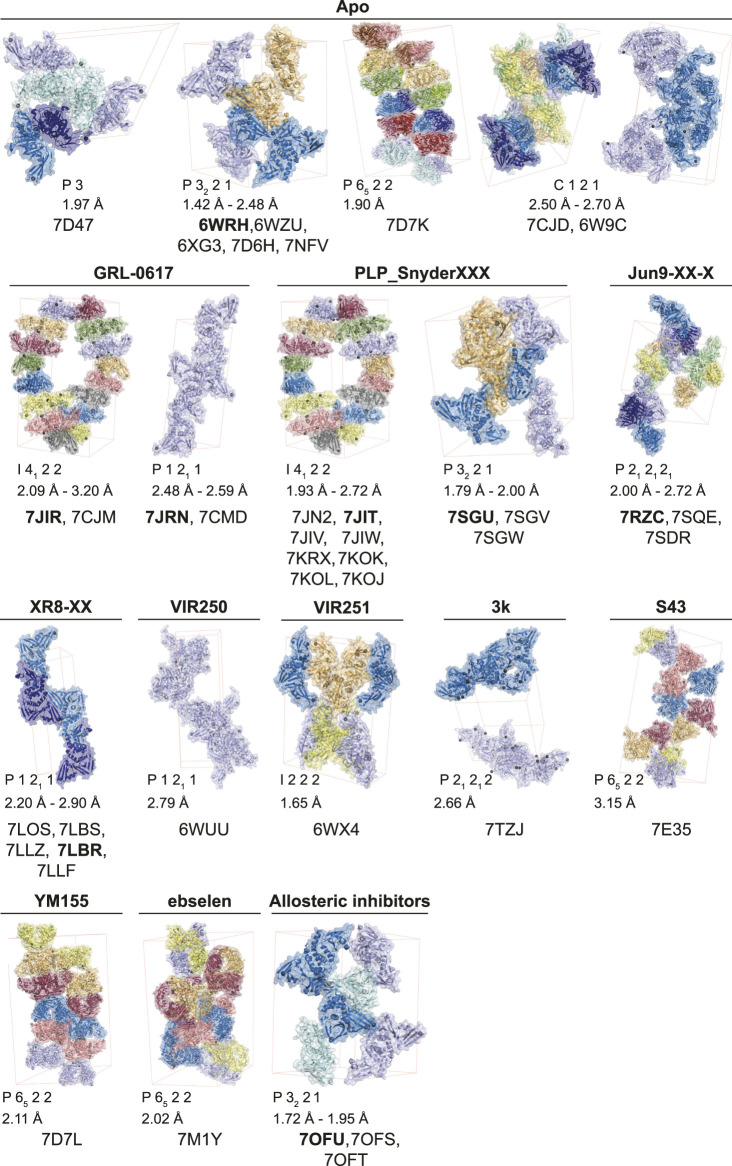
Reported apo and compound structures of SARS-CoV-2 PLpro. Shown are unliganded and compound bound structures publicly released in the Protein Data Bank (PDB) since the beginning of the COVID-19 pandemic. The unit cell for each space group is shown (thin red lines) and the corresponding symmetry mates from the asymmetric unit are depicted with matching colours. The structures are grouped according to their bound ligands, the ligand is labelled above each unit cell, and the corresponding PDB accession numbers shown below. The obtained resolution or resolution range for each crystallographic setting is indicated.

**FIGURE 2:**
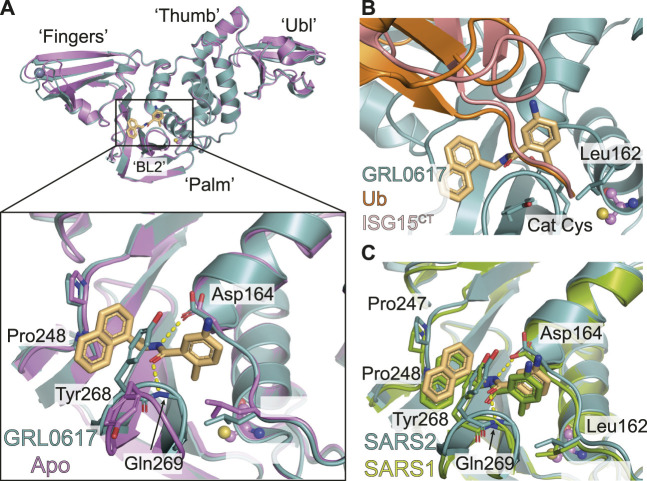
Molecular basis for inhibition of SARS-CoV-2 PLpro by *GRL-0617*. **(A)** Structure of SARS-CoV-2 PLpro bound to ***GRL-0617*** in teal (PDB 7JRN (Ma et al., 2021)), with inhibitor in wheat colour in ball-and stick representation representing the (R)-enantiomer. A close-up view of the ligand binding site for ***GRL-0617*** with key residues indicated is also shown and hydrogen bonds are displayed as a dashed yellow line. A superimposed structure of apo PLpro [purple, PDB 6WZU (Osipiuk et al., 2021)] shows that the inhibitor does not induce global conformational changes. The catalytic Cys is shown in ball and stick representation, and a bound zinc ion in apo PLpro is shown as a grey sphere. **(B)** Close-up view of the ***GRL-0617*** binding site overlaid with ubiquitin from the ubiquitin-PLpro complex in orange [PLpro ∼ Ub, PDB 6XAA (Klemm et al., 2020)] and with ISG15 from PLpro bound to the C-terminal ISG15 Ubl fold in pink [PLpro ∼ ISG15^CT^, PDB 6XA9 (Klemm et al., 2020)]. The orthomethyl resides in a pocket formed by Leu162, Tyr264, and Tyr273 occupying the position of the Arg74 in Ub or Arg155 in ISG15. Upon ligand binding, Leu162 rotates its side chain to block the channel and the path of the Ubl tail to the catalytic Cys111. The catalytic Cys111 is shown in ball and stick representation. **(C)** Close up view of the ligand binding site for ***GRL-0617*** in SARS-CoV-2 PLpro in teal overlaid with SARS-CoV PLpro in green [PDB 3E9S (Ratia et al., 2008)]. Key residues are fully conserved between SARS-CoV and SARS-CoV-2 which explains cross specificity of compounds. Hydrogen bonds are displayed as a dashed yellow line.

The start of the pandemic triggered by a previously unknown pathogen, has led to an unprecedented response of the structural biology community that focused on the proteins of SARS-CoV-2 with all available tools and techniques. As a result, structures of the SARS-CoV-2 proteome have flooded the PDB. The first crystal structures of PLpro were those bound to mono-ubiquitin (PDB 6XAA) (Klemm et al., 2020), the C-terminal domain of human ISG15 (ISG15^CTD^, PDB 6XA9) (Klemm et al., 2020), full length mouse ISG15 (PDB 6YVA) (Shin et al., 2020), as well as high resolution apo structures [PDB IDs 7D47, 7NFV, 6W9C–unpublished, 6WRH, 6WZU, 6XG3 (Osipiuk et al., 2021), 7D6H (Shan et al., 2021), 7D7K (Zhao et al., 2021) and 7CJD (Gao et al., 2020)]. The first inhibitor bound structures utilised peptide-based inhibitors, VIR250 (PDB 6WUU) (Rut et al., 2020) and VIR251 (PDB 6WX4) (Rut et al., 2020). Collectively, these apo- and substrate-bound structures were instrumental to provide comprehensive insight into PLpro function and mechanism, but also highlighted the rather high similarity between SARS-CoV and SARS-CoV-2 PLpro. Notably, SARS-CoV-2 PLpro appears to be highly amenable to crystallisation, and there are to date 14 distinct crystal settings (different space groups and/or unit cell dimensions) for PLpro and its complexes (Figure 1). Indeed, the majority of by-now available structures of SARS-CoV-2 PLpro, are complex structures with bound inhibitors, especially from the ***GRL-0617*** class (Figures 1, 2). Elsewhere in this issue, we report a structure of SARS-CoV-2 PLpro bound to inhibitor ***3k*** explaining intricacies of the piperidine carboxamide based inhibitors, ***5c*** and ***3k***. This series of compounds were reviewed early in the pandemic (Ghosh et al., 2020) and we discuss our medicinal chemistry efforts geared towards addressing metabolic liabilities of these compounds.

Definition and exploitation of the ***GRL-0617*** binding pocket or ‘hot spot’ (Fu et al., 2021), which is shared with ***3k/5c***-class compounds, has substantially benefited from structure-guided drug design. New reports focussing on optimising ***GRL-0617*** for its binding site are published frequently, and the current state-of-play is reviewed in the next sections.

### Origin of *GRL-0617*

HTS campaigns performed by the team of Andrew Mesecar against SARS-CoV PLpro, followed by extensive medicinal chemistry led to the development of ***GRL-0617*** (Ratia et al., 2008; Ghosh et al., 2009), a SARS-CoV inhibitor with sub-μM activity *in vitro* that inhibited SARS-CoV viral replication in cell-based infection studies. The mechanism of inhibition was explained *via* a co-crystal structure (PDB 3E9S) (Ratia et al., 2008), highlighting how the compound targeted the binding channel required to interact with the cleavage motif. Researchers quickly realised that identical residues line the ligand binding sites in SARS-CoV-2 PLpro, and it was no surprise that ***GRL-0617*** also inhibited viral replication of SARS-CoV-2 (Freitas et al., 2020; Shin et al., 2020). These results further cemented PLpro as an excellent drug target for COVID-19 antiviral treatments. Subsequent structures of SARS-CoV-2 PLpro in complex with ***GRL-0617*** (Gao et al., 2020; Fu et al., 2021; Osipiuk et al., 2021) confirmed that the binding site and mode of inhibition, as a reversible competitive inhibitor, was virtually identical to that for SARS-CoV PLpro.

## Overview of the PLpro Inhibitor Binding Site

***GRL-0617*** binds to a groove within the ‘Palm’ domain of PLpro (for nomenclature Figure 2A), used to hold the cleavage motif of PLpro. However, the most prominent aspect of its binding mechanism relies on a flexible segment, termed blocking loop 2 (BL2) (Lee et al., 2015), a β-hairpin that folds over the core of the compound and shields it from solvent. Tyr268 at the tip of the β-hairpin restrains the substituted benzamide, almost entirely burying it in the enzyme (Figure 2A). On one side of the compound, the naphthyl ring extends into a hydrophobic groove between BL2 and the Palm domain, packing against Pro247 and Pro248. Two hydrogen bonds further stabilise the compound in its binding site; the amide nitrogen of the compound with the side chain of Asp164 of the “Thumb” domain; and the amide carbonyl of the compound with the backbone of Gln269 on BL2 (Figure 2A). Towards the catalytic Cys111, some 7 Å away, an orthomethyl group on the substituted phenyl ring fits into a hydrophobic pocket lined by Leu162, Tyr264 and Tyr273 (Figures 2A,B), occupying the position of the usually positively charged residue (Lys or Arg) preceding the Gly-Gly motif (including Arg74 in ubiquitin or Arg155 in ISG15; hereafter, we refer to the ubiquitin residue numbers). Leu162 that lines the channel in apo and substrate bound structures, rotates its side chain to block the channel and the path to catalytic Cys111, and now interacts with the substituted phenyl of the compound (Figures 2A,B) (see peptide inhibitors below for an example of where Leu162 indeed rotates again to open the congested channel). This conformational change is seen in all ***GRL-0617*** or ***5c*** compound structures to date and is a good indicator of compound binding.

As discussed above, the binding mode for ***GRL-0617*** to SARS-CoV-2 PLpro could be anticipated due to high structural and sequence identity with SARS-CoV PLpro. Indeed, the interacting residues and all compound interactions are conserved between SARS-CoV and SARS-CoV-2 PLpro (Figure 2C). The work in the last 2 years elaborated the vicinity of the GRL scaffold, mainly in order to improve on the observed IC_50_ of ∼1–2 µM *in vitro* (Table 2 for a list of current studies, their HTS assay(s) and observed IC_50_ of *GRL-0617*). Since ***GRL-0617*** was already the result of extensive medicinal chemistry, most researchers attempted to achieve potency increase by expanding the ***GRL-0617*** core.

**TABLE 2:** Currently reported potencies (IC_50_, µM) of *GRL-0617* against SARS-CoV-2 PLpro.

IC_50_ (µM)	Primary assay	References
1.15	Ub-Rh110	Calleja et al. this issue
0.74	Ub-AMC	Shin et al. (2020)
1.50	ISG15-AMC	
0.88	Ub-AMC	Ma et al. (2021)
1.68	ISG15-AMC	
1.39	Z-RLRGG-AMC	Zhao et al. (2021)
1.61	Z-RLRGG-AMC	Shen et al. (2021)
2.1	Z-RLRGG-AMC	Fu et al. (2021)
2.2	(Dabcyl)-FTLRGGAPTKV-(Edans)	Gao et al. (2020)
2.3	LKGG-(CV-2)	Osipiuk et al. (2021)
2.4	Z-RLRGG-AMC	Freitas et al. (2020)

### Targeting Glutamate 167 (Glu167)

**FIGURE 3:**
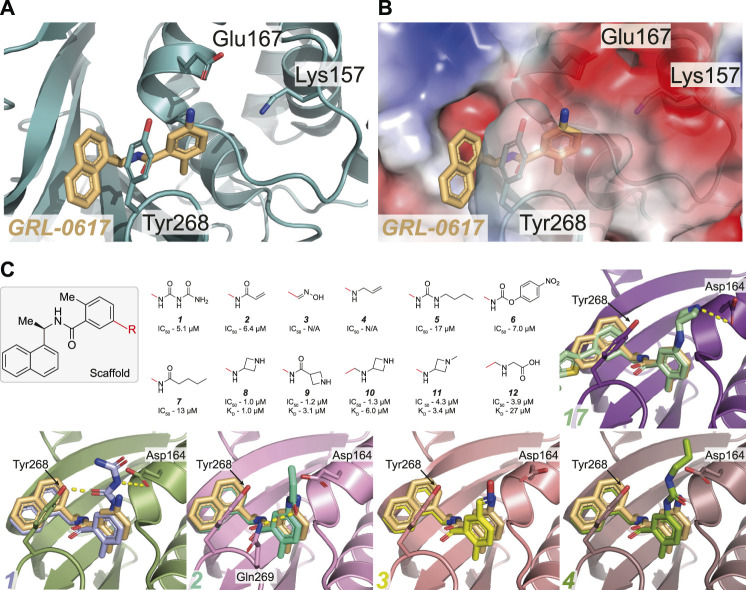
Overview of the reported attempts to target Glu167 in PLpro. **(A,B)** Close up view of the ***GRL-0617*** binding pocket with PLpro in teal in cartoon representation **(A)** or as with the calculated surface charge overlaid **(B)**. The inhibitor is shown in wheat colour in ball-and stick representation. Residues Lys157 to Glu167 in the PLpro Thumb domain form a shallow negatively charged pocket. Several compounds target the side chain of Glu167 to improve potency of ***GRL-0617***. **(C)** The ***GRL-0617*** scaffold (boxed) was extended at the para-position of the benzene ring (at the R position). The red line indicates the handle used for the respective substituents - in ***GRL-0617*** an amino group is present at this position. Given compound data refer to, IC_50_ from *in vitro* activity assays, and K_D_ values from SPR where available (See Table 3 for the compound identifiers from their respective publications). Basic amines appear to be most tolerated at this position while replacement with alkyl groups are detrimental to activity of the compound. Co-crystal structures for ***1–4*** and ***17*** (Figure 4) are shown [PDB IDs ***1*** (7JIT), ***2*** (7JIW), ***3*** (7KOL), ***4*** (7KOJ), ***17*** (7LBS)]. Compounds ***3*** and ***4*** were published in the PDB but excluded from the final publication, so no IC_50_ data is available (Osipiuk et al., 2021).

The first crystal structures of ***GRL-0617*** bound to SARS-CoV-2 PLpro reveal that the orientation of the steric clash between the orthomethyl group on the benzene ring and the amide group forces the methyl into an orientation that mimics the substrate backbone of ubiquitin Arg74 (Figures 2B, 3A). In this orientation, the charged Glu167 side chain is solvent exposed and within reach by expanding from the 5-amino group towards (Figures 3A,B). Prior SAR on SARS-CoV PLpro indicated that changes to this site were tolerated (Ghosh et al., 2009; Welker et al., 2021) and hence several groups have since attempted to expand from this handle.

One of the earliest groups to design compounds targeting Glu167 (***1–7,*** Figure 3C and Table 3) was at the Centre for Structural Genomics of Infectious Diseases (CSIG), and reported co-crystal structures for four compounds (1–4, Figure 3C) (Osipiuk et al., 2021). In ***1***, a carbonyl group creates additional hydrogen bonds with Glu167 (3.0 Å bond distance) and Tyr268 hydroxyl from the BL2 loop (2.4 Å), whereas an acrylamide moiety in ***2*** adopts a different conformation, forming a H-bond interaction with the side chain of Gln269 (3.2 Å). The remaining compounds (***3–4***) make no new interactions (Figure 3C). Although ***1*** and ***2*** both have additional contacts with PLpro, they incur a > 2-fold loss in potency observed in comparison with ***GRL-0617*** (Table 2) suggesting that the new interactions do not enhance the stability of the complex. Similarly, all other compounds reported in (Osipiuk et al., 2021) (***5–7***, Figure 3C), did not improve on ***GRL-0617***.

**TABLE 3:** Compound IDs from their respective studies for those reported in Figures 2–5.

Review ID	Reference ID	IC_50_ (µM)	Primary assay	References	Review ID	Reference ID	IC_50_ (µM)	Primary assay	References
1	Snyder_495 (2)	5.1	LKGG-(CV-2)	Osipiuk et al. (2021)	20	XR8-89 (94)	0.11	Z-RLRGG-AMC	Shen et al. (2021)
2	Snyder_530 (3)	6.4			21	XR8-69 (89)	0.37		
3^a^	Snyder_496	—			22	XR8-23 (72)	0.39		
4^a^	Snyder_494	—			23	XR8-32-1 (75)	0.97		
5	5	17			24	XR8-30 (74)	0.75		
6	6	7			25	DY-3-63 (18)	>100		
7	7	13			26	ZN-2-193 (21)	>10		
8	ZN-2-184 (5)	1.01	Z-RLRGG-AMC	Shen et al. (2021)	27	ZN-2-192 (20)	4.8
9	ZN-2-186 (7)	1.2			28	Jun9-13-7	7.3	(Dabcyl)-FTLRGGAPTKV-(Edans)	Ma et al. (2021)
10	DY2-144 (14)	1.3			29	Jun9-13-9	6.7		
11	ZN-2-188–2 (11)	4.3			30	Jun9-53-2	0.89		
12	ZN-3-56 (13)	3.9			31	Jun9-72-2	0.67		
13	ZN-3-80 (65)	0.59			32	Jun9-87-3	0.80		
14	XR8-8 (66)	1.3			33	Jun9-87-2	0.90		
15	ZN-3-79 (59)	1.9			34	Jun9-87-1	0.87		
16	DY-2-153 (60)	1.8			35	Jun9-75-5	0.56		
17	XR8-24 (73)	0.56			36	Jun9-84-3	0.67		
18	XR8-65 (86)	0.33			37	Jun9-75-4	0.62		
19	XR8-83 (92)	0.21			38	Jun9-85-1	0.66		

a **3** and **4** structures were published in the PDB, prior to publication of (Osipiuk et al., 2021), but excluded from the final publication. The two compounds were presented in this Review to illustrate their structural features.

**FIGURE 4:**
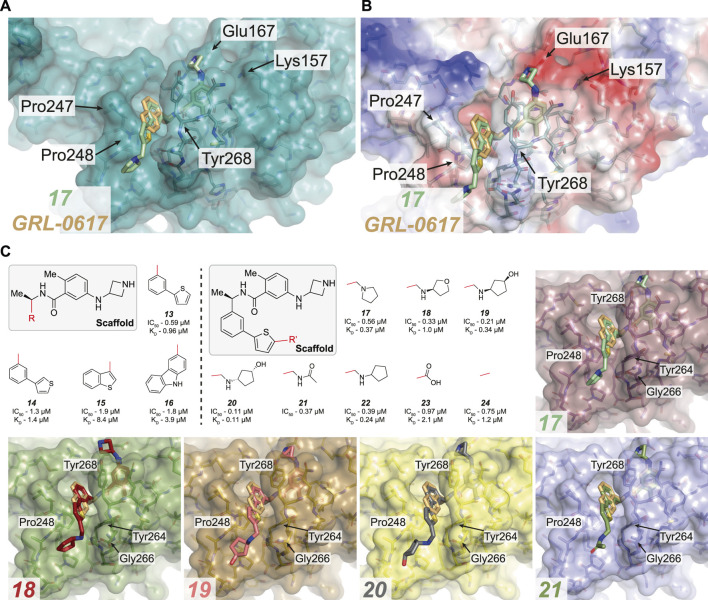
Overview of compounds which successfully replaced the naphthyl ring in ***GRL-0617***. **(A,B)** Close up view of the ***GRL-0617*** binding pocket with PLpro represented in teal in cartoon representation overlaid with the surface representation **(A)** or calculated surface charge of the protein **(B)**. ***GRL-0617*** in wheat colour and compound ***17*** in green are depicted in ball and stick representation. In **(A)** the key residues are noted to highlight the BL2 groove formed by closure of the blocking loop and induced upon ligand binding. ***17*** shows that the replacement of the naphthalene ring with a 2-phenylthiophene appears to effectively replace the dependency of the naphthyl group. The BL2 groove is then engaged by a basic amine tail to improve potency. **(C)** Boxed (left), the parent scaffold for modifying the naphthyl group. Boxed (right) Second iteration of compound designs, starting at the 2-phenyl thiophene scaffold (***13***). The red line indicates the handle used for the respective substitution. Compound data refer to IC_50_ from *in vitro* activity assays, and K_D_ values from SPR where available (see Table 3 for the compound identifiers from their respective publications). ***13–16,*** the 2-phenyl thiophene appears to successfully replace the naphthyl ring while still maintaining potency. ***17–24***, Aromatics containing basic amines appear to be the most potent at this position. Co-crystal structures for ***17–21*** are shown [PDB IDs, ***17*** (7LBS), ***18*** (7LOS), ***19*** (7LLF), ***20*** (7LBR), ***21*** (7LLZ)]. In the case of ***17***, the basic nitrogen interacts with residues lining the BL2 groove.

Another series of compounds (Shen et al., 2021) observed the variable activity of extensions towards Glu167 (***8–12,*** Figure 3C). The direct addition of an azetidine ring to the 5-amino group of ***GRL-0617*** in ***8*** provided the most potent and successfully increased the potency of ***GRL-0617*** from 1.6 to 1.0 µM (Table 2; Figure 3C). A modelled structure of ***8*** highlighted a potential interaction between Glu167 and the azetidine nitrogen, a prediction then confirmed by a crystal structure of a related compound, from the same study (***17*** *,* Figure 3C) (Shen et al., 2021). In ***17***, the naphthyl ring is replaced with a 2-phenylthiophene scaffold (Figure 4), the effects of which are described in the next section.

Alternative analogues prepared by Osipiuk et al. (2021) also engaged Glu167, but none of them were as potent as the azetidine containing compounds reported by Shen et al. (2021). Azetidine nitrogens are more basic than amides, ureas, anilines, or carbamates used by Osipiuk et al., which may explain the marked difference in efficacy. Compounds ***8***, ***9,*** and ***10*** display a narrow IC_50_ range of 1.0, 1.2 and 1.3 µM (Figure 3C), respectively, but differing only in their positioning of the H-bond acceptor. Removing the H-bond acceptor entirely in this position resulted in a 4-fold loss of potency (compounds ***11–12***) ***.*** It is interesting to note that these subtle changes in protein-ligand interactions appear more pronounced when comparing the K_D_ values of these compounds obtained by SPR (1.0, 3.1 and 6.0 µM respectively, Figure 3C).

It is clear from Osipiuk et al. (2021) and Shen et al. (2021), that targeting Glu167 in isolation is unlikely to provide significant improvement in activity required to justify *in vivo* studies. Nevertheless, both studies provided valuable structural information about PLpro compound binding.

### Targeting the BL2 Groove and the Naphthyl Ring

The naphthyl ring in ***GRL-0617***, a moiety crucial for its activity, packs in a tight hydrophobic pocket of PLpro (Figures 4A,B). The original SAR for naphthyl ring subsitutions was performed for SARS-CoV PLpro: both empirical and computational methods confirmed its replacements obliterate compound activity (with the 1-naphthyl being preferred over the 2-naphthyl) (Ghosh et al., 2009; Amin et al., 2021; Welker et al., 2021). Notwithstanding its contribution to the binding affinity of ***GRL-0617*** to PLpro, naphthyl groups come with many liabilities (outlined below), so it is not surprising that many groups have attempted to find more suitable and druglike isosteres.

Naphthyl moieties exist in clinically used drugs in a broad range of diseases, but their presence must be carefully considered as they can add significant metabolic liabilities and are often viewed as toxicophores (Makar et al., 2019). In addition, they significantly increase lipophilicity of a compound. Shen et al. (2021) combined their designs targeting Glu167 with additional changes to the naphthyl ring (***13–16,*** Figure 4C and Table 3), achieving sub-µM efficacy *in vitro* (***17–24,*** Figure 4C and Table 3). Their most successful replacement was a 2-phenylthiophene scaffold, which notably leveraged binding cooperativity when combined with the azetidine ring targeting Glu167 (compare ***8*** in **Figure 3C** and ***13*** in Figure 4C). Interestingly, further substitutitions to this scaffold were tolerated (***17–24,*** Figure 4C) and those with a basic amine “tail” were favoured (***17–22,*** Figure 4C).

Several structures were reported (***17–21***), and each highlighted that the novel scaffold targeted a previously unexplored hydrophobic “BL2 groove” found adjacent to the blocking loop; (Shen et al., 2021). intruiguingly, all but compound ***17*** showed disordered ‘tail’ regions in BL2 groove, suggesting that it remains highly flexible. It might be possible that each of the “tail” regions are trapped by a network of transient interactions with residues lining the BL2 groove (Gly266, Pro248 and Tyr264) that together contribute to an overall lower free energy state, possibly explaining the observed mobility and disorder within the crystal structures. In the case of ***17***, the basic nitrogen interacts with residues lining the BL2 groove - including the amide backbones of Tyr264 and Tyr268 (Figure 4C), and explains the observed improvement in potency when compared to the thiophene scaffold alone (compare ***13***). ***17*** also exhibited a ∼5-fold lower dissociation rate (K_D_) and improved antiviral efficacy over ***GRL-0617***. It was exciting to see the improved antiviral efficacy accompanied by the replacement of the naphthyl group. Replacing this compound moiety is a promising development on this class of inhibitors as it likely represents a metabolic liability along the path to the clinic.

### A Tertiary Amine Connects GRL-0617 Based Compounds to Asp164

**FIGURE 5:**
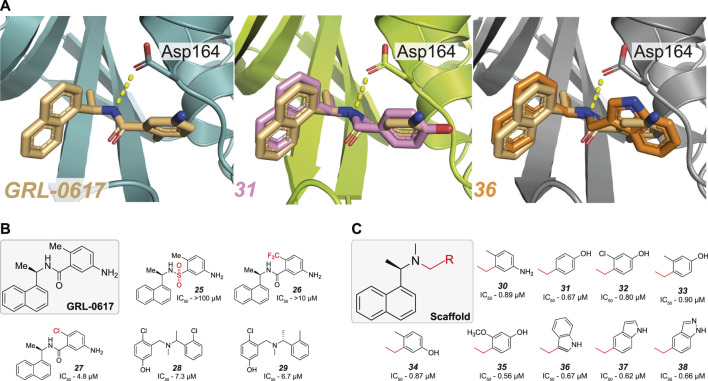
Modification of the amide bond in *GRL-0617*. **(A)** Close up view of the binding pocket for ***GRL-0617, 31*** (PDB 7SDR) and ***36*** (PDB 7RZC)**.** PLpro is represented in teal, lime green or grey respectively in cartoon representation and the inhibitors are shown in wheat, pink, or orange respectively in ball-and-stick representation. In ***GRL-0617*** the side chain of Asp164 forms a crucial H-bond interaction with the amide nitrogen and the orthomethyl group remains invariant in most derivatives, as its binding pocket restricts the orientation of the phenyl ring. In ***31*** and ***36*** the amide bond was successfully replaced with a tertiary amine and both compounds appear to have effectively removed the dependency on the orthomethyl group by increasing the bond strength with Asp164. **(B)** The amide bond and orthomethyl appear to be highly sensitive to variation (***25–27***). Conservative substitution with a chlorine group (***27***) reduces the IC_50_ 5-fold, to 4.8 µM. Hit compounds ***28***–***29*** from (Ma et al., 2021) enabled the merging of compound properties with GRL-0617. **(C)** Compounds explored in (Ma et al., 2021). A tertiary amine enables more extensive variations to the phenyl group not achieved prior, while retaining compound potency.

Another delicate interaction formed between PLpro and ***GRL-0617*** is a network of hydrogen bonds between the central carboxamide, the side chain of Asp164 and the backbone nitrogen of Gln269 (Figures 2A, 5A). Various attempts have failed to replace the central amide in ***GRL-0617*** (Shen et al., 2021; Welker et al., 2021) where the isosteric change to a sulphonamide group also dramatically reduced activity (***25***, Figure 5B and Table 3) (Shen et al., 2021). In ***GRL-0617***, the amide is juxtaposed by the orthomethyl substituent found on the phenyl ring amide (Figure 5A) mimicking the backbone of ubiquitin Arg74. The orthomethyl is invariant in all iterations of ***GRL-0617*** mentioned thus far, and compound activity is extremely sensitive to changes at this site (Ghosh et al., 2009; Shen et al., 2021). For example, changing the methyl for a trifluoro-methyl (***26***, Figure 5B and Table 3), a larger group with different electronegativity, ablated compound activity, whereas exchanging the methyl with a chlorine group (***27***, Figure 5B and Table 3) decreases potency by ∼5-fold (Shen et al., 2021).

Another recent HTS campaign (Ma et al., 2021), identified two hit compounds (***28–29,*** Figure 5B and Table 3) which differed from ***GRL-0617*** in that the hits were lacking the naphthyl ring and the central amide was replaced with a tertiary amine. Furthering their efforts, the team were able to merge the properties of all three compounds to achieve sub µM efficacy is many of their optimised compounds (***30***–***38***, Figure 5C). In ***GRL-0617,*** the carboxamide carbonyl forms a connection with the backbone nitrogen of Gln269 (Figure 2A), a connection presumably lost when replaced with a tertiary amine. In their new compounds, the tertiary amine would likely be protonated in physiological conditions - exposing a positively charged nitrogen instead of the neutral NH of the amide. Interestingly, the protonated tertiary amine appeared to substitute for the loss of the carboxamide connection, as ***30*** still retained equipotent activity to ***GRL-0617***. Structures from two of their optimised compounds, were later released by the CSIG (***31, 36***, Figure 5A, PDB 7SDR and 7RZC, respectively) confirming that the amine indeed formed a more prominent H-bond with the side chain of Asp164—the carboxyl group of which rotates slightly to optimise the interaction (Figure 5A, compare ***GRL-0617***).

Another important insight from this new scaffold was that it allowed for a greater diversity of substitutions on the phenyl group, alleviating the need for an orthomethyl group. Similar potency was achieved with compounds where the *ortho*-position was either unsubstituted (***31*** *,* Figure 5C), substituted with chlorine (***32*** *,* Figure 5C) or larger groups (***33***–***35*** *,* Figure 5C) and further, the entire phenyl could be replaced with an indole group (***36***–***38*** *,* Figure 5C). Hence, the idea to replace the amide bond with a tertiary amine appears to have unlocked a useful new scaffold for SAR exploration.

The above examples highlight the progress of the scientific community in elaborating a decade-old PLpro inhibitor, ***GRL-0617***, through iterative medicinal chemistry. While not discussed here, many of the applied design principles may also guide improvements for other PLpro inhibitor series, in particular the piperidine scaffolds exemplified by compounds ***5c*** and ***3k*** (see Table 1). Whilst not achieved to date, we are confident that low nM inhibitors for PLpro, likely required for meaningful clinical translation, are within reach.

### An Alternative Strategy: Covalent Peptide Inhibitors

A common strategy to target Cys proteases is to identify and then permutate peptide-based inhibitors that directly target the catalytic Cys. While peptide inhibitors are challenging as drugs due to metabolic liabilities, susceptibility towards amide bond hydrolysis and poor cell penetration, they are a mainstay for medicines mimicking protein-protein interactions (PPIs) (Lau and Dunn, 2018). This strategy has worked recently for Mpro, and is the basis for the now-FDA approved covalent Mpro peptidomimetic inhibitor from Pfizer, Nirmatrelvir (Owen et al., 2021). An important aspect was that Mpro is exquisitely specific for hydrolysing substrates directly after a glutamine residue, a property not seen in any human cysteine protease, alleviating cross-specificity and toxicity concerns. This is somewhat more of a problem for PLpro, since the existing PLpro target preference for the LXGG (Rut et al., 2020) motif is present in ∼100 DUBs and other ubiquitin-like proteases in the human genome.

Yet, the Olsen group generated competitive, covalent peptide inhibitors for PLpro from a combination of natural and unnatural amino acids (Rut et al., 2020). The peptides, dubbed VIR250 and VIR251, were effective inhibitors across multiple CoV species however, their specificity against human Ubl proteases were not reported. Peptide inhibitors may prove useful in deciphering the required residues that dictate specificity through the protease active site, and the co-crystal structures obtained (PDB 6WUU, 6WX4) contributed early-on to the detailed characterisation of SARS-CoV-2 PLpro.

To improve on the efficacy of such inhibitors for PLpro, there have been recent attempts at combining the specificity of small molecules (such as ***GRL-0617***) with the potency of covalent peptides or war heads (Liu et al., 2021a; Parks et al., 2021). In particular the latter manuscript, currently available as a preprint, discusses how ***GRL-0617*** is derivatised to reach the catalytic Cys111, and a crystal structure shows that Leu162 indeed rotates again to open the congested channel typically observed in liganded PLpro structures. It is interesting to note that PLpro retains this plasticity, though it appears that other compounds (***2*** in Figure 3C) are unable to invoke this conformational change despite incorporating potential covalent war heads.

This approach to synthesize peptide-drug conjugates (PDCs) targeting SARS-CoV-2 PLpro appeared to improve on specificity toward the catalytic cysteine. Yet, these PDCs were still found to be nonselective for the other ten cysteines found in PLpro (Liu et al., 2021a). While peptide-based inhibitors remain an interesting avenue for development, the issues of their specificity for PLpro, and typically low oral bioavailability indicate that, as for Nirmatrelvir (Owen et al., 2021), significant medicinal chemistry will be required to convert the peptidic features into more favourable drug-like properties.

### Other Identified Small Molecule Inhibitors of PLpro

There are several other studies that identified small molecule inhibitors of PLpro that are here mentioned for completeness. One study identified the Survivin inhibitor YM155 (Zhao et al., 2021), and while a structure bound to PLpro was released (PDB 7D7L), it appears that no direct interactions are taking place to indicate this is a true PLpro inhibitor. Further, the side chain from the crucial Tyr268 residue, claimed to embrace the compound in a similar fashion to ***GRL-0617***, remains unresolved in the submitted structure. In addition, YM155 was also found to be cytotoxic in a recent follow up study (Ma and Wang, 2022).

Another study identified a well-known pan DUB inhibitor, PR-619 (Liu et al., 2021b) and the USP1 inhibitor SJB2-043 as direct inhibitors of PLpro. Both highlight the similarity of PLpro to human DUBs, and may be interesting tools for *in vitro* experiments, though ***GRL-0617*** seems a superior tool at this point. Others also identified 6-thioguanine and 6-mercaptopurine (Sivakumar and Stein, 2021; Swaim et al., 2021), which were later invalidated as either inactive or toxic in follow-up cellular assays (Ma and Wang, 2022). Finally, a number of naturally occurring compounds were also highlighted (Srinivasan et al., 2021) for their activity as allosteric inhibitors of PLpro (preprint at the time of writing this Review). Structures of these (Figure 1) highlighted that they inhibit substrate binding at the S2 site of PLpro, a feature not yet seen for any PLpro inhibitors. Allosteric inhibitors are a largely unexplored avenue for targeting PLpro and it remains unclear how effective such inhibitors would be in the context of inhibiting full length Nsp3. While new insights may be gleaned from e.g., structural work with non-specific compounds in principle, all mentioned compounds seem very far away to warrant clinical development.

## Conclusion and Outlook

We have witnessed the unprecedented rise of a global pandemic caused by the lethal coronavirus SARS-CoV-2. To date, it is estimated that COVID-19 has killed almost 6 million people worldwide. However, the response from the scientific community has also been unprecedented, in scale, speed and collaborative spirit. Many researchers have refocussed their efforts to better understand, and eventually help defeat, SARS-CoV-2, and we have witnessed a striking number of incredible scientific achievements, first-and-foremost a global vaccination effort based on latest technological achievements. In addition, the pandemic has propelled to the forefront, and shown the immense value of, areas of basic research that were considered niche only a few years ago. Indeed, the achievements described here were building on a rich well of prior knowledge, provided by a small number of research labs that have studied earlier coronaviruses for decades, and whose work has identified cell biological and biochemical mechanisms, validated and de-risked viral targets, and provided essential starting points to make quick progress in drug discovery.

It is clear that antiviral drugs for COVID-19 remain one of the most pressing necessities to regain normality after the pandemic. The first antivirals have recently emerged and will quickly become key tools for clinicians treating COVID-19 patients. However, it can also be safely assumed that SARS-CoV-2 will find a way to alleviate this new attack, and the emergence of drug resistance mutants is just a matter of time. For this and other reasons, our efforts to develop new antivirals, for SARS-CoV-2 and ideally all CoVs, need to continue and require long-term support and funding.

In our minds, PLpro is the prime untapped target for the next CoV antiviral medicine. It is, by now, well-studied and understood, essential for CoV lifecycle, and its moonlighting functions as DUB and deISGylase derail our cellular inflammatory responses, a hallmark of the most marked pathologic outcomes of COVID-19. This latter function as a DUB, presents challenges and opportunities. On one hand, specific DUB inhibitors are notoriously challenging to develop, and have to date only been achieved for a handful of DUBs out of the pool of ∼100 human enzymes. For example, the USP7 specific inhibitors, FT671 and FT827, each relied on pockets not found in the apo or substrate bound forms of the enzyme, and which were induced upon ligand binding (Turnbull et al., 2017). This feature is also observed for the current PLpro inhibitors which target the BL2 “hot spot” and though the scaffolds targeting this site had been relatively limited prior to the pandemic, the studies mentioned in this Review have highlighted possibilities to generate novel chemical scaffolds. Only one DUB inhibitor has entered clinical trials to date. Surprisingly and despite high structural conservation of PLpro required to cleave specific sequences, to date the identified inhibitors seem all SARS-CoV and SARS-CoV-2 specific and do not target MERS-CoV (most other CoV PLpros have not been assessed). It needs to be seen whether a pan-CoV PLpro inhibitor is achievable.

Nonetheless, as we detail in this Review, armed with prior knowledge from SARS-CoV and in just 2 years we have seen rapid developments to advance a promising inhibitor scaffold, based on ***GRL-0617*.** Further increases in potency are paramount to enter lead optimisation, and then a detailed assessment and improvement of pharmacokinetics or pharmacodynamics is required. Such studies will be important contributions for the advancement of PLpro inhibitors to the clinic. Considering the speed of discovery and scale of theglobal effort, we expect to see breakthroughs on the ***GRL-0617*** series, the related piperidine-based ***5c*** series, and/or on as yet unreported compound series originating from fresh HTS campaigns, later in 2022.
